# Epidemiology, genetic diversity and symptom characterization of *rotavirus* infection in Saudi Arabia, 1985–2024: a meta-analysis

**DOI:** 10.3389/fmicb.2025.1671607

**Published:** 2025-10-21

**Authors:** Khalid J. Alzahrani

**Affiliations:** Department of Clinical Laboratories Sciences, College of Applied Medical Sciences, Taif University, Taif, Saudi Arabia

**Keywords:** *rotavirus*, genotype, epidemiology, vaccination, Saudi Arabia

## Abstract

**Introduction:**

*Rotavirus* infection has been a major health burden among children under 5 years in Saudi Arabia. There is a lack of meta-analysis on epidemiology and genetic diversity of *rotavirus* in Saudi Arabia.

**Methods:**

We conducted this study to provide a comparative overview of *rotavirus* infection in Saudi Arabia. We selected published literature between 1985 and 2025.

**Results:**

Epidemiological data were retrieved from 18 articles. In Saudi Arabia, the pooled prevalence of RVA among children under 5 years was 34.3% (95% CI: 2% to 81%, I^2^ = 98.97%). Overall, G1P[8] (52%, 95% CI: 29% to 69%, I^2^ = 0%) was reported from the highest number of cases, followed by G2P[4] (18%, 95% CI: 9% to 31%, I^2^ = 36.86%), G9P[8] (14%, 95% CI: 8% to 27%, I^2^ = 45.37%), G12P[8] (4%, 95% CI: 2% to 11%, I^2^ = 0%), and G3P[8] (3%, 95% CI: 1% to 11%, I^2^ = 99%), respectively. Genotype G2P[4] (41%) became the most prevalent, while the frequency of G1P[8] reduced to 38% and G9P[8] to 6% during 2013–2024. The odds of *rotavirus* infection increased in the winter season (aOD 2.4, 95% CI: 1.52 to 3.57, *p*-value 0.005). The odds of *rotavirus* prevalence were significantly lower after vaccination (aOD 0.56, 95% CI: 0.25 to 0.73, *p*-value 0.001).

**Discussion:**

This is one of the first meta-analyses to compare the genotypic data of *rotaviruses* before and after vaccination in Saudi Arabia. This study will provide an overall insight into prevalence, genetic diversity, and seasonality during pre-vaccination and post-vaccination periods and contribute to policy making.

## Introduction

*Rotavirus* is the main (family *Reoviridae*) pathogen of gastroenteritis among children under 5 years worldwide (Aliabadi et al., 2019; Kotloff et al., 2017; Milivojevic and Milosavljevic, 2020). Every year, approximately 300,000 deaths associated with *rotavirus* infection are documented globally (Aliabadi et al., 2019). *Rotavirus*-associated deaths of under-five children have been partially reduced due to vaccination (Coşkun and Kasap, 2019; Hartman et al., 2019; Kotloff, 2017). The emergence of new genotypes and partial coverage of vaccines are still contributing to the high morbidity of *rotavirus*-associated gastroenteritis (Dennehy, 2015; Kheyami et al., 2006). Globally, *rotavirus* is one of the major causes of childhood morbidity and mortality. The health burden of *rotavirus* among children is also reflected in the WHO Eastern Mediterranean Region, with higher cases and mortality than other diarrheal pathogens. The estimated incidence of *rotavirus* is 7000 cases per 100,000 children under 5 years in Saudi Arabia. According to the WHO, severe and prolonged health conditions are reported from about 25%–30% of *rotavirus* cases (Kheyami et al., 2006; Troeger et al., 2018).

*Rotavirus* is a double-stranded (dsRNA, segmented), non-enveloped, RNA virus with an 18.55 kilobase pair *genome* (Estes and Cohen, 1989). The *genome* encodes six structural (VP1 to VP6) and six non-structural proteins (NSP1 to NSP6) (Estes and Cohen, 1989; Lefkowitz et al., 2018). Among ten species, *rotavirus* A is the major human pathogen. *Rotaviruses* are classified into different genotypes, including the G genotype based on glycoprotein (VP7) and the P genotype based on protease (VP4) (Estes and Cohen, 1989; Lefkowitz et al., 2018). Approximately 36 G-genotypes and 51 P-genotypes are circulating globally (Lefkowitz et al., 2018; Patton, 2012). *Rotavirus* genotypes G2P[4], G1P[8], G3P[8], G9P[8] and G4P[8] are the most widely documented worldwide. Among the *rotavirus* P-genotypes, P[4], P[6], and P[8] are the most prevalent globally (Al-Aidaroos et al., 2017; Ashwan et al., 2012; Kawai et al., 2012; Miles et al., 2012; Patton, 2012; Sharif et al., 2023). *Rotavirus* genotypes G1 to G4 were the most prevalent before 2000, and genotypes G9 and G12 became widespread after 2000 in Asia and the Middle East (Lefkowitz et al., 2018; Patton, 2012; Shaheen, 2019). The genotypic diversity in Saudi Arabia is also high for *rotavirus* infection, and it has changed over the recent years (Al-Aidaroos et al., 2017; Ashwan et al., 2012; Kawai et al., 2012; Miles et al., 2012; Page et al., 2021; Shaheen, 2019; Sharif et al., 2023).

Saudi Arabia is a temperate country with higher air temperatures and aridity. It occupies around 80% of the Arabian Peninsula and has a population of 34 million (Al-Aidaroos et al., 2017; Ashwan et al., 2012). *Rotavirus*-associated diarrhea is still a notable health issue in Saudi Arabia. Previous studies have reported the prevalence of *rotavirus* among diarrheal children between 8% to 65% in Saudi Arabia (Kawai et al., 2012; Patton, 2012; Shaheen, 2019; Sharif et al., 2023). Though the incidence has reduced in some regions, the overall prevalence of *rotavirus* is still high and underestimated.

The WHO prequalified and internationally accepted *rotavirus* A vaccines, including RotaTeq (bovine-human reassorted vaccine), and Rotarix (human *rotavirus*), are available in Saudi Arabia. Since 1 January 2013, *rotavirus* vaccines have been included in the national immunization program in Saudi Arabia (Kheyami et al., 2006; Troeger et al., 2018). Though *rotavirus* vaccination has been included in the national immunization program in Saudi Arabia for the last 13 years, meta-data analysis on the genotypic diversity of *rotavirus* is lacking. Knowing the changing epidemiology and genetic diversity of circulating *rotavirus* can contribute to the evaluation of the effectiveness of available vaccines. The main aim of this study is to determine the prevalence and genotypic diversity of *rotavirus* in Saudi Arabia.

## Methods

### Definitions

This study included data from the previously published epidemiological, virological, and clinical research on *rotavirus* in Saudi Arabia. The positive case was confirmed by the ELISA-based method before the invention of the RT-PCR laboratory-confirmed test. The prevalence of *rotavirus* was defined as the proportion of the study population with a *rotavirus*-positive test at a specific study. We defined the epidemiology of *rotavirus* as the distribution and determinants of the cases in study populations. The transmission was defined as the spread of the virus from the sources to the susceptible individuals. We followed the standards of the Preferred Reporting Items for Systematic Review and Meta-Analysis (PRISMA) Statement and Cochrane Collaboration to perform this study (Page et al., 2021).

### Case definition

For defining a confirmed *rotavirus* case, we followed the case definition by the WHO and the final classification guideline. We included the data from the previously published paper confirming the presence of *rotavirus* by reverse transcriptase polymerase chain reaction (PCR)-based method or an enzyme immunoassay (EIA) method. The specific class of RT-PCR was also determined following the local guidelines and availability of the methods in a particular study population.

### Study design

The study was conducted by adopting different strategies. At first, the search strategies and specific objectives were determined, followed by finding appropriate research articles, setting inclusion and exclusion criteria of manuscripts, data collection, quality assessment, screening, data analysis, inclusion of the findings, summarization, and exclusion of irrelevant data. The inclusion criteria consist of several factors, including original research articles, prevalence data, genotypic data, epidemiological data, case studies, outbreak investigation, surveillance work, hospital-based surveillance, case-control studies, and online databases. All of the previous studies on the selected topic missed the parameters for the quality assessment. Hence, the quality reports by the authors were the major source of data for this study.

### Search strategy and selection criteria

The relevant data were collected from articles published in peer-reviewed journals. Data were retrieved from Web of Science, Scopus, MEDLINE (through PubMed), EMBASE, ASM journals, The New England Journal of Medicine (NEJM), Cell Press journals, Journals from Saudi Arabian Publishers, and The Lancet with no restriction on language. This study included data from only Saudi Arabia. All published peer-reviewed manuscripts till January 01, 2025, were included in this study.

The search terms included Saudi Arabia, *Rotavirus*, Human *Rotavirus*, Infection, Epidemiology, Coinfection, RVA, RVB, RVC, Symptoms, Seasonality, Genotypes, Vaccines, Children, Gastroenteritis, Rapid kit, RT-PCR, Diarrhea, 2000 to 2025, Clinical features, Signs and Symptoms, Cases, Clinical characteristics, Transmission, Prevention, Food-born illness, Water-borne illness, Molecular epidemiology, Genetic diversity, Infants, ELISA, WHO, CDC, Hospitals, Clinics, Morbidity, Mortality, Prevalence, Incidence, Age, Gender, Sex, Population, Acute gastroenteritis and several combination of these terms. Combined and separate searches were performed with these terms on every website, journal page, and database.

Additionally, the Google Scholar database and gray literature were also searched to get all the possible study links. The relevant data were searched in WHO (the World Health Organization), CDC (Centers for Disease Control and Prevention, USA), ProMed, ECDC (European Centers for Disease Control and Prevention), Epicenter, and local health surveillance websites in Saudi Arabia. This study also analyzed and screened data from the yearly update of health data. Preprint databases, namely bioRxiv, SSRN, medRxiv, and AAS Open Research, were searched for any relevant data. In addition, the first thirty pages on the Google Scholar search engine were searched for the search terms. This study integrated the epidemiologic data of *rotavirus* outbreaks by cumulating studies on incidence, case reports, prevalence over the years, genetic diversity, clinical history, seasonality, geographic distribution, and vaccination.

The author conducted the quality evaluation of selected studies. The repeated and partial data were removed after searching the data sources, journals, published articles, and websites. The modeling of outbreaks and prediction studies, review articles, editorials, correspondences, mini-reviews, book chapters, environmental factors studies, and non-relevant studies to the objectives were excluded. Further, the author performed a critical quality evaluation of the selected manuscripts, identification of duplicated articles, and removal of correspondence or comment on duplicated data multiple times. Articles containing the relevant data were included for all geographic locations in Saudi Arabia, all ethnicities, children under 18 years, all sexes, and clinical features. A full-text analysis of the selected articles was conducted.

The risk of bias in the selected manuscripts was measured using the JBI tool. This JBI tool used nine parameters to assess the bias of the selected studies (Munn et al., 2015). Each parameter was evaluated using the findings as not applicable, unclear, yes, or no. Further, the author also used the Systematic Review Center for Laboratory Animal Experimentation (SYRCLE) assessment tool where applicable (Zeng et al., 2015). The SYRCLE tool consists of 10 parameters for assessing various biases. The parameters included selection bias, reporting bias, detection bias, attrition bias, performance bias, and other biases. The bias for the selected parameter was measured by following the previously published scales using the possible results as yes, no, and unclear, for low, high, and unclear bias, respectively (Zeng et al., 2015).

The data on meteorological factors were collected from different servers and websites, including the official website of the Saudi Arabia Meteorological Department^1^, Weather Forecast Saudi Arabia^2^, AccuWeather^3^ meteoblue^4^, and WeatherOnline^5^. The sociodemographic factors were taken from the included studies.

### Statistical analysis

Descriptive statistical analysis, including frequencies, percentages, mean, and standard deviation (SD), was calculated for selected data of rotavirus-positive cases. The pooled data were reported as proportions with 95% confidence intervals (CI). The heterogeneity of the data was assessed by the I^2^-test. Values <25% were recorded as mild, 25%–75% as intermediate, and >75% as significant heterogeneity. The overall pooled prevalence was determined using the DerSimonian-Laird method (Zhao, 2013). Forest plots were used to represent genotype-specific pooled prevalence. The pooled prevalence and genotypic variation were determined by using SAS version 9.4. Linear logistic regression analysis was conducted to find out the impact of environmental factors and sociodemographic factors on the odds of *rotavirus* infection.

## Results

### Included studies

**FIGURE 1:**
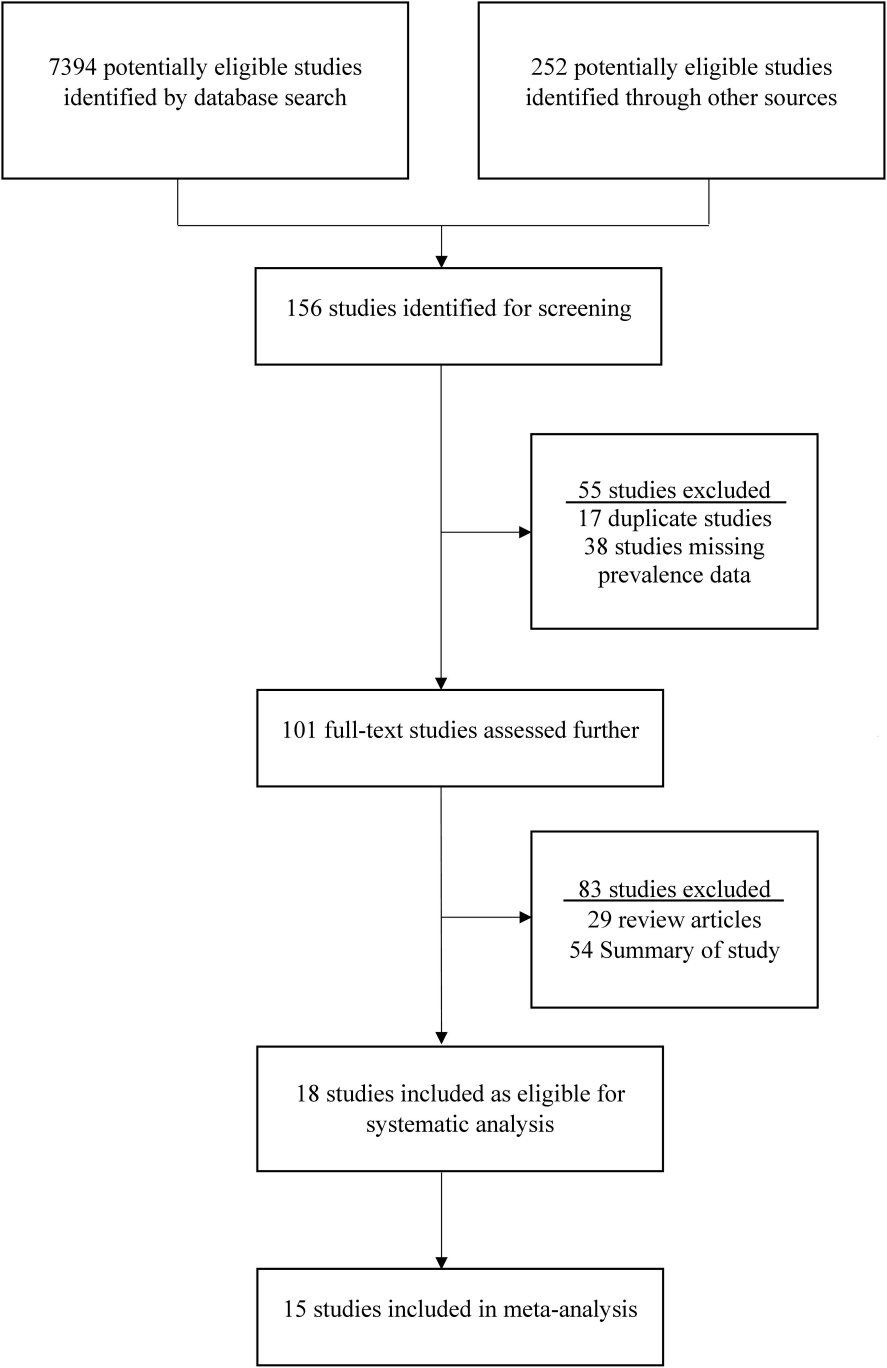
The procedure of selection and screening of studies on *rotavirus* in Saudi Arabia. The standards of the Preferred Reporting Items for Systematic Review and Meta-Analysis (PRISMA) Statement was followed to identify and select the eligible studies.

This study found 7394 previous articles and 252 documents on the search topics in Saudi Arabia. After the first screening, only 156 articles were considered for analyzing the abstracts. From these 156 articles, 55 were excluded based on the exclusion criteria, and a full-text analysis was conducted on 101 articles. After that, 83 articles were excluded from these 101 articles, and 18 were selected for the systematic review (Afifi and Nabiha, 2013; Al-Ayed et al., 2017; Almalki et al., 2022; Alqurayn et al., 2024; Aly et al., 2015; Eifan et al., 2023; El Assouli et al., 1996; Ghazi et al., 2005; Hegazi et al., 2017; Khalil et al., 2015; Kheyami et al., 2008; Meqdam and Thwiny, 2007; Obeid, 2011; Paul et al., 2021; Qadri et al., 1990; Tayeb et al., 2008, 2011; Zaki et al., 2017; Zhao, 2013). Among the 18 selected articles, epidemiological data were taken from 18 articles, prevalence data from 18 articles, clinical symptoms from 10 articles, and genotyping from 7 studies (Figure 1). Finally, 15 studies were included in the meta-analysis, and the other 3 studies were excluded due to the lack of data for meta-analysis.

### Prevalence of *rotavirus* infection in Saudi Arabia

The prevalence of *rotavirus* A (RVA) in Saudi Arabia has been underreported during the last 40 years. This study found 18 original manuscripts reporting the prevalence of *rotavirus* from 1985 to 2025. In Saudi Arabia, the pooled prevalence of RVA among children under 5 years was 34.3% (95% CI: 2% to 81%, I^2^ = 98.97%). Among these 18 studies from 1985 to 2025, RVA was reported in 18 (100%) studies. These studies confirmed the presence of RVA by ELISA (100%) or RT-PCR methods. This study found 100% of the *rotavirus* cases from hospital surveillance in Saudi Arabia. The higher prevalence of RVA (40% of the gastroenteritis) was documented before 2013. After the start of vaccination in 2013, the prevalence of RVA dropped significantly (from 40 to 8%) among the children in the study regions. The majority of the cases were reported from children under 2 years (81%, 95% CI: 58% to 93% I^2^ = 65.98%). All of the cases (100%) were from *rotavirus* A (Table 1).

**TABLE 1:** Prevalence and epidemiology of *rotavirus* infection in Saudi Arabia.

References	Study period	No of the study participants	Age of the participants	Prevalence% (*N*)	Diagnosis method	*Rotavirus* species
Zhao, 2013	Mar 1984 to Mar 1985	150	<5 years	30%(45 of 150)	ELISA	RVA
Qadri et al., 1990	1987 to 1988	859	<5 years	37.5% (240 of 859)	ELISA	RVA
El Assouli et al., 1996	Apr 1992 to Feb 1993	349	<2 years	43%(150 of 349)	ELISA	RVA
Ghazi et al., 2005	Jan 2003 to Dec 2003	459	<5 years	10%(48 of 459)	ELISA	RVA
Meqdam and Thwiny, 2007	2004 to 2005	284	<5 years	33.1% (94 of 284)	EIA	RVA
Kheyami et al., 2008	Apr 2004 to Apr 2005	984	<5 years	19%(187 of 984)	ELISA	RVA
Tayeb et al., 2008	Sep 2002 to Aug 2003	1000	<5 years	6% (60 of 1000)	ELISA, RT-PCR	RVA
Tayeb et al., 2011	Jan 2008 to Oct 2010	1007	<5 years	65.5% (660 of 1007)	ELISA	RVA
Obeid, 2011	2007 to 2008	156	<5 years	23.7% (37 of 156)	ELISA, STAT-PAK test	RVA
Afifi and Nabiha, 2013	Jan 2010 to Dec 2010	301	<5 years	34%(101 of 301)	ELISA	RVA
Aly et al. (2015)	2011 to 2012	541	<2 years	31.6% (171of 541)	ELISA, RT-PCR	RVA
Khalil et al., 2015	Feb 2007 to Mar 2008	970	<5 years	40.7% (395 of 970)	ELISA, RT-PCR	RVA
Al-Ayed et al., 2017	Oct 2013 to Sep 2015	850	<5 years	9.2% (78 of 850)	ELISA, RT-PCR	RVA
Zaki et al., 2017	Sep 2011 to Aug 2012 and Sep 2015 to Aug 2016	730	<16 years	26.4% (193 of 730)	SD BIOLINE *rotavirus* kits	RVA
Hegazi et al., 2017	Oct to Dec 2015	359	<5 years	3.9% (14 of 359)	I_*G*_A	RVA
Paul et al., 2021	Nov 2017 to Jan 2019	307	<5 years	6.5% (20 of 307)	Rapid Card InstaTest, RT-PCR	RVA
Eifan et al., 2023	Feb 2017 to Jan2018	100	<5 years	2% (2 of 100)	RT-PCR	RVA
Alqurayn et al., 2024	Jan 2000 to Dec 2022	580	<14 years	13.6% (79 of 580)	LFA	RVA

RVA, *rotavirus* A; ELISA, enzyme-linked immunosorbent assay; RT-PCR, reverse transcriptase polymerase chain reaction (conventional); LFA, lateral flow assay; EIA, enzyme immunoassay.

### Distribution of *rotavirus* cases in Saudi Arabia

**FIGURE 2:**
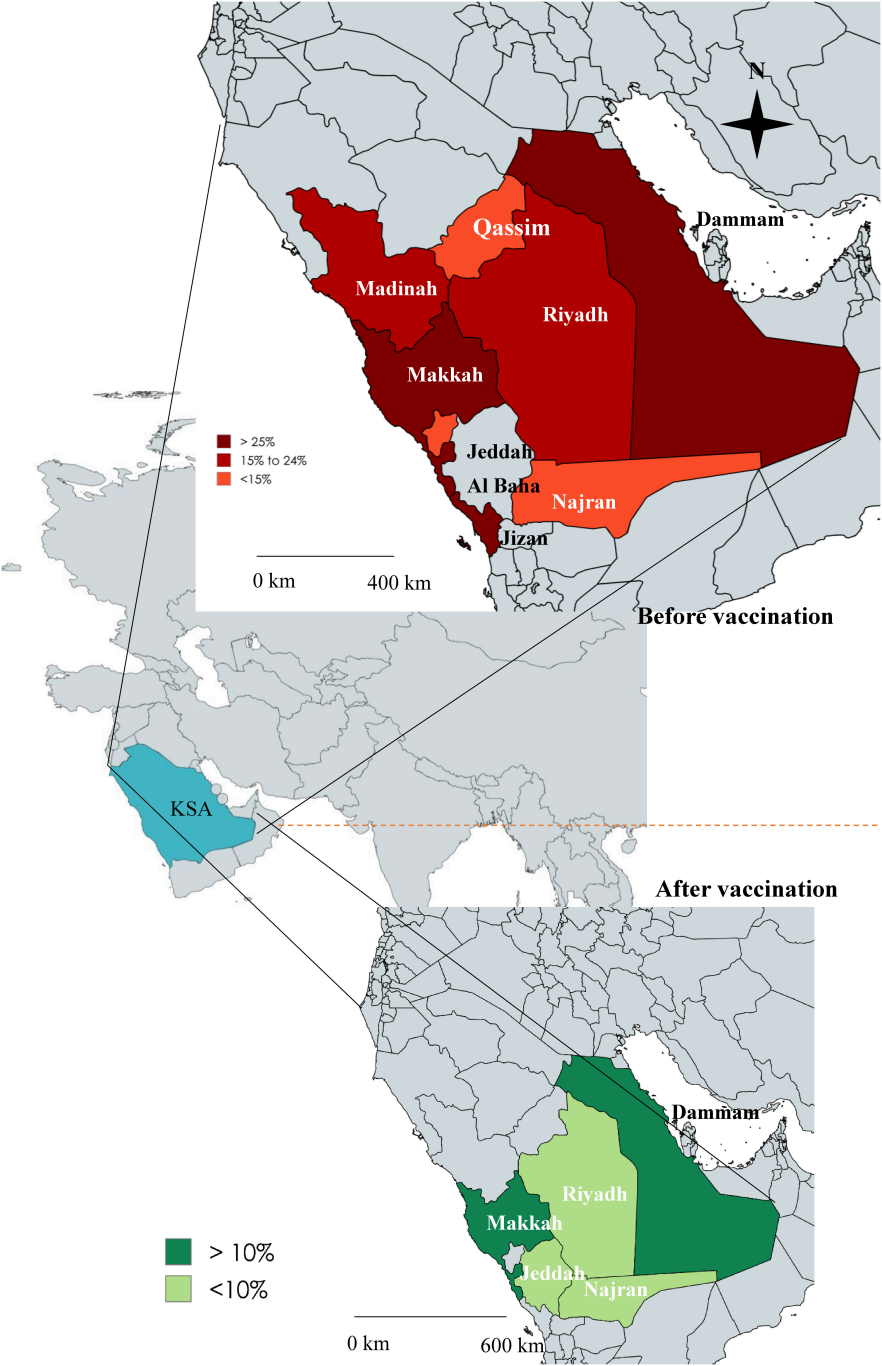
Map of Saudi Arabia showing the comparative prevalence of *rotavirus* in different regions before vaccination (upper panel) and after vaccination (lower panel).

*Rotavirus* infection has been reported from the majority of areas in Saudi Arabia. The first report of confirmed cases was from Dammam during 1984–85, with a higher prevalence of 30% positive children. A higher prevalence of RVA (26%, 95% CI: 24% to 48%, I^2^ = 2.34%) was reported from Dammam (1987–88), Taif (1992–93), Makkah, Jeddah, and Jizan, as well as from Dammam (2002–10). The prevalence of RVA ranged from 15 to 24% from 2007 to 2013 in Riyadh and 15% in Qassim and Taif of Saudi Arabia. In Dammam and Makkah, the prevalence of RVA was 14% (95% CI: 12% to 24%, I^2^ = 0%) after the initiation of vaccination in 2013. In Riyadh, Jeddah, and Najran, the prevalence of RVA was reduced below 10% (95% CI: 1% to 9%, I^2^ = 0%) from 2014 to 2024 (Figure 2).

### Genetic diversity of *rotavirus* A in Saudi Arabia

**FIGURE 3:**
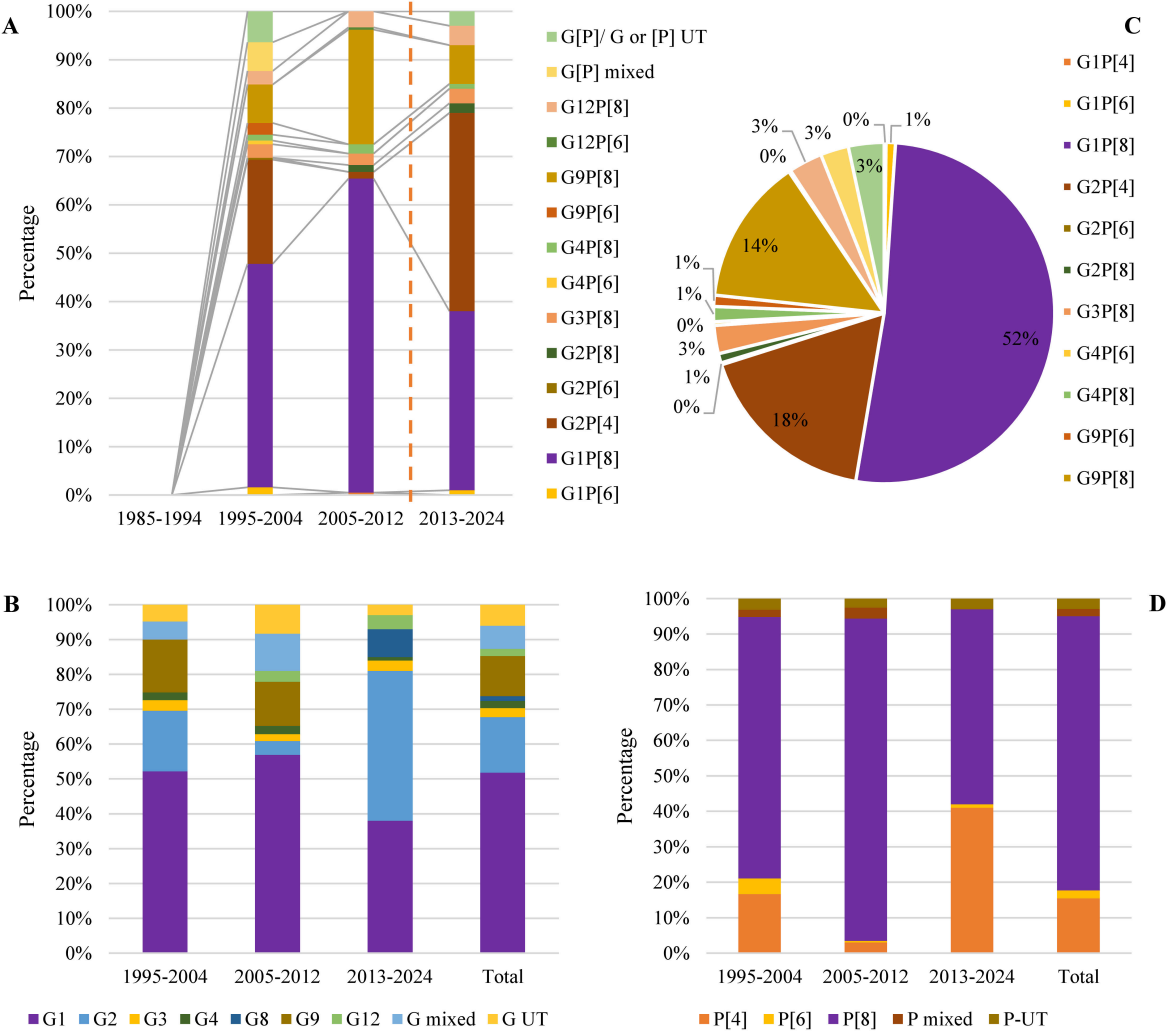
**(A)** Year-wise genotypic distribution of *rotavirus* in Saudi Arabia, **(B)** proportionate frequency of different genotypes of *rotavirus* in Saudi Arabia, **(C)** Year-wise prevalence of G-type, *rotavirus* and **(D)** Year-wise prevalence of P-type *rotavirus*. Before vaccination is represented by data from 1995 to 2012, and after vaccination is represented by data from 2013 to 2024.

The studies on the genetic diversity of RVA were less frequent in Saudi Arabia. We found 39% (7 of 18) studies reported the genotypic information. *Rotavirus* G-typing and P-typing were conducted for 583 isolates. Genotype G1P[8] (48%, 95% CI: 26% to 62%, I^2^ = 1.39%) was the most prevalent, followed by G2P[4] (21%, 95% CI: 13% to 39%, I^2^ = 0%), G9P[8] (9%, 95% CI: 2% to 14%, I^2^ = 76.87%), G3P[8] (3%), and G12P[8] (3%), respectively, during 1995–2004. Genotype G1P[8] (67%, 95% CI: 36% to 79%, I^2^ = 0%) remained the most prevalent and G9P[8] (24%, 95% CI: 15% to 31%, I^2^ = 8.93%) replaced G2P[4] (2%) during 2005–2012. Genotype G2P[4] (41%, 95% CI: 26% to 48%, I^2^ = 2.34%) became the most prevalent from 2013 to 2024, while the frequency of G1P[8] reduced to 38% (95% CI: 12% to 51%, I^2^ = 97.45%) and G9P[8] to 6% (Figure 3).

**FIGURE 4:**
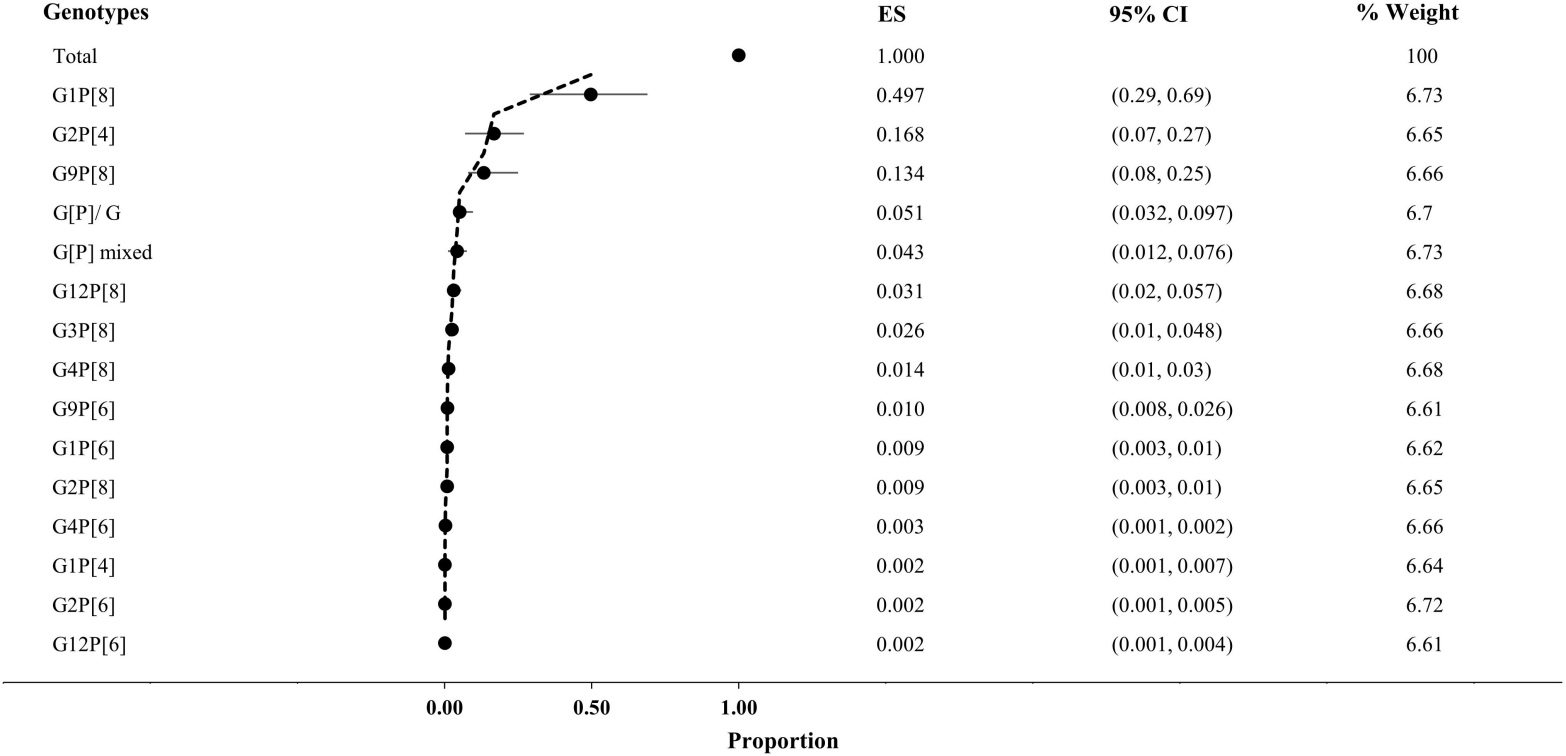
Prevalence of genotypes of *rotavirus* in Saudi Arabia. CI, confidence interval; ES, effect size (proportion of genotypes); Weight, inverse variance.

Overall, G1P[8] (52%, 95% CI: 29% to 69%, I^2^ = 0%) was reported from the highest number of cases, followed by G2P[4] (17%, 95% CI: 7% to 27%, I^2^ = 36.86%), G9P[8] (13%, 95% CI: 8% to 25%, I^2^ = 45.37%), G12P[8] (3%, 95% CI: 2% to 6%, I^2^ = 0%), and G3P[8] (3%, 95% CI: 1% to 5%, I^2^ = 99%), respectively (Figure 4). About 4% of the isolates were non-typable, and 3% were mixed. Among the other reported genotypes, G1P[4], G1P[6], G2P[6], G2P[8], G4P[6], G4P[8], G9P[6], and G12P[6] were found in <2% frequency in Saudi Arabia (Figure 3).

Among the 583 G-typed isolates, G1 (52%, 95% CI: 29% to 77%, I^2^ = 0%) was the most frequently reported, followed by G2 (19%, 95% CI: 8% to 32%, I^2^ = 32.67%) and G9 (11%, 95% CI: 4% to 25%, I^2^ = 98%), respectively, from Saudi Arabia. During 1995–2012, G1 (55%) was the most prevalent, followed by G9 (17%), and the prevalence of G2 reduced from 18% to 3% from 1995–2004 to 2005–12. After the start of vaccination in 2013, G2 (42%) became prevalent, followed by G1 (37%) and G9 (11%), respectively (Figure 3).

Among the 583 P-typed isolates, P[8] (78%, 95% CI: 36% to 89%, I^2^ = 0%) was the most frequently reported, followed by P[4] (17%, 95% CI: 11% to 25%, I^2^ = 26.21%) and P[6] (3%, 95% CI: 2% to 8%, I^2^ = 0%), respectively, from Saudi Arabia. During 1995–2012, P[8] (87%) was the most prevalent, followed by P[4] (10%). After the start of vaccination in 2013, the prevalence of P[4] (42%) increased significantly (*p*-value 0.005), and the prevalence of P[8] reduced from 87 to 55% (Figures 3, 4).

Different genotypes of *rotavirus* were widely distributed in several regions in Saudi Arabia. Genotype G1P[8] was reported from almost every region included in this study, with a higher prevalence than other genotypes. Genotype G2P[4] was reported in the children with diarrhea from Riyadh, Aseer, Jeddah, Dammam, Jizan, and Makkah.

### Seasonality of *rotavirus* A in Saudi Arabia

**FIGURE 5:**
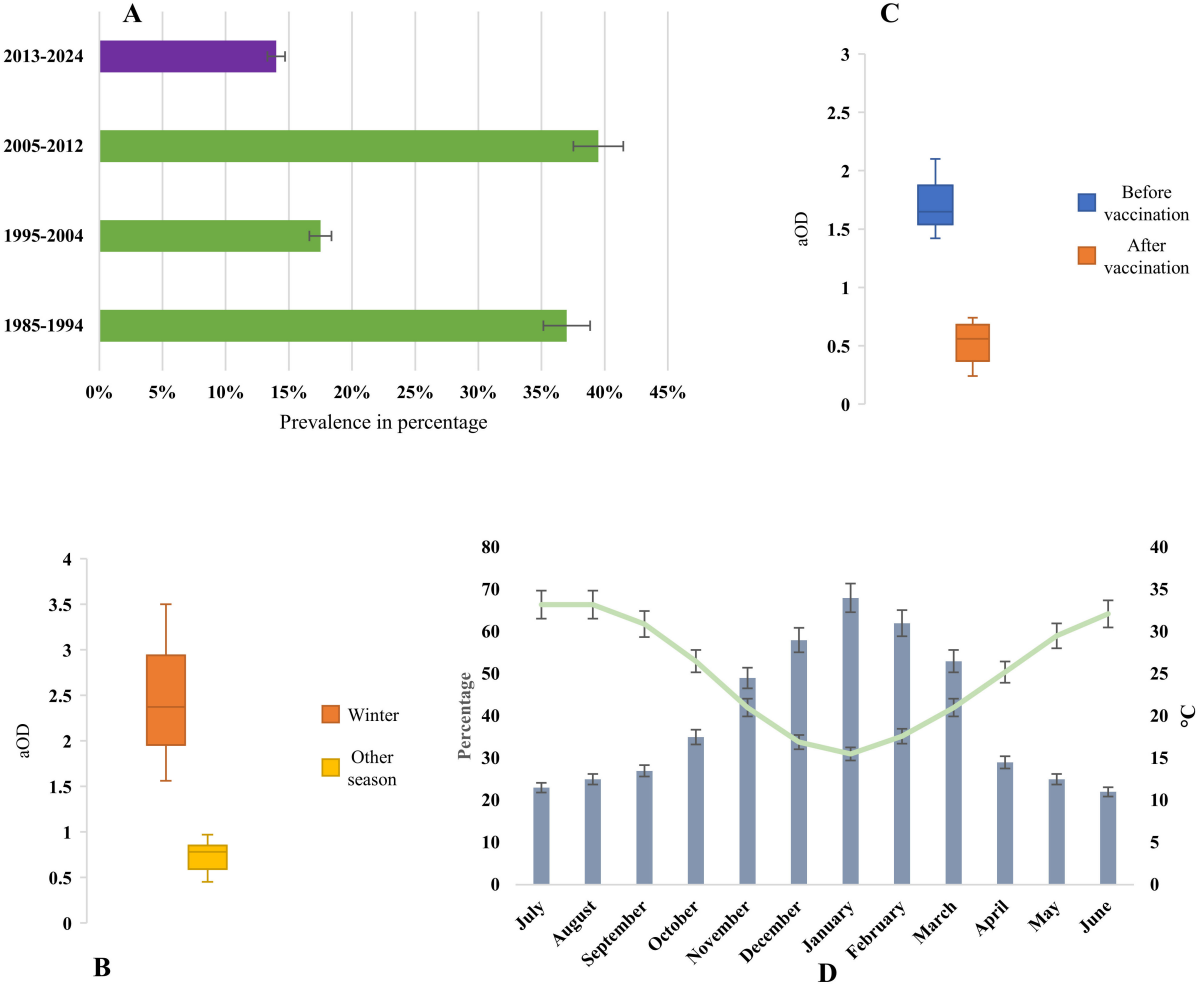
**(A)** Year-wise prevalence of *rotavirus* infection in Saudi Arabia, before vaccination is represented by data from 1995 to 2012, and after vaccination is represented by data from 2013 to 2024. **(B)** Adjusted odds ratio of seasonality of *rotavirus*, **(C)** Adjusted odds ratio of prevalence of *rotavirus* after and before vaccination, and **(D)** Seasonal prevalence of *rotavirus* and changes of average day temperature in Saudi Arabia.

The highest prevalence of *rotavirus* was reported during 2005–2012 (39%), followed by 1985–1994 (37%), 1995–2004 (17%), in the pre-vaccination era. However, after the vaccination program, the prevalence reduced significantly to 14% during 2013–2024 (Figure 5). The data from 2013 to 2024 indicated post-vaccination periods, and from 1985 to 2012 represented pre-vaccination periods. The data on *rotavirus* seasonality were extracted from 11 studies during 1985–2024 in Saudi Arabia (Figure 5). *Rotavirus* infection was reported all year round in Saudi Arabia. The odds of *rotavirus* infection increased in the winter season (aOD 2.4, 95% CI: 1.52 to 3.57, *p*-value 0.005) with an average temperature of 16 °C. We also analyzed the odds of the prevalence of *rotavirus* infection after vaccination. The odds of *rotavirus* prevalence were significantly lower after vaccination (aOD 0.56 95% CI: 0.25 to 0. 73, *p*-value 0.001). The highest peak of *rotavirus* infection (70%, 95% CI: 50%–78%, *p-*value 0.001) was reported from November to March in Saudi Arabia (Figure 5). The pattern of the seasonality of *rotavirus* was consistent in the majority (89%) of the studies.

### Clinical characteristics of *rotavirus* infection

Data on the clinical presentations of *rotavirus*-infected children were retrieved from 10 studies. About 1162 *rotavirus*-positive children under 5 years showed different symptoms in Saudi Arabia. Diarrhea (86%, 95% CI: 65% to 96%, *p*-value 0.01) was the most common symptom followed by dehydration (79%, 95% CI: 45% to 86%, *p*-value 0.05), vomiting (74%, 95% CI 52% to 78%, *p*-value 0.03), fever (68%, 95% CI: 39% to 74%, *p*-value 0.01) and abdominal pain (37%, 95% CI: 33% to 64%, *p*-value 0.05), respectively. About 100% (10 of 10) of the studies reported diarrhea followed by dehydration (90%, 9 of 10), vomiting (90%, 9 of 10), and fever (80%, 8 of 10), respectively. *Rotavirus*-positive children had a higher risk of developing severe diarrhea (aOR: 2.54, 95% CI: 1.64 to 2.84, *p-*value 0.05), dehydration (aOR: 2.59, 95% CI: 1.82 to 3.19, *p-* value 0.005), and severe vomiting (aOR: 1.76, 95% CI: 1.04 to 2.13, *p-*value 0.02) (Table 2). We found that children aged below 2 years (aOR: 3.36, 95% CI: 2.59 to 3.76, *p-*value 0.05) had significantly higher odds of *rotavirus* infection, and the sex of the children was not associated with the incidence of *rotavirus* in Saudi Arabia.

**TABLE 2:** Adjusted odds ratio of symptoms among the *rotavirus* positive children.

Symptoms	aOR	95% CI	*P*-value
Diarrhea	3.21	2.43–4.15	0.005
Severity of diarrhea	2.54	1.64–2.84	0.05
Vomiting	2.26	1.57–2.76	0.001
Frequency of vomiting per 8 h	1.76	1.04–2.13	0.02
Fever	3.54	2.15–3.79	0.01
Severity of fever	1.02	0.41–1.45	0.03
Abdominal pain	1.59	1.14–2.54	0.05
Nausea	2.18	1.35–2.85	0.01
Dehydration	2.59	1.82–3.19	0.005

aOR, adjusted odds ratio; CI, confidence intervals. The total study population was 3464 for the analysis of adjusted odds ratio of symptoms among the 1162 *rotavirus* positive children under 5 years. The odds ratio of symptoms were adjusted for age, sex and places in the model.

## Discussion

*Rotavirus* infection was one of the major causes of child morbidity in Saudi Arabia (Aleid et al., 2024; Almalki et al., 2022; Alqurayn et al., 2024; Eifan et al., 2023; Paul et al., 2021; Troeger et al., 2018). *Rotavirus* vaccination has been included in the national immunization program since 2013, and Saudi Arabia has one of the highest vaccine coverages for *rotavirus* (97%) globally (Troeger et al., 2018). However, studies on the genetic diversity, epidemiology, seasonality, and clinical characteristics of *rotavirus* infection are lacking in Saudi Arabia. We conducted this study to provide a comparative insight into *rotavirus* infection before and after vaccination in Saudi Arabia. First, this is one of the early studies of meta-analysis of *rotavirus* before and after vaccination in Saudi Arabia. The pooled prevalence of *rotavirus* infection was 34.3% (95% CI: 2% to 81%, *p*-value 0.001). However, we found high heterogeneity in the prevalence data across different studies. There might be several reasons, including some of the studies reporting pre-vaccination data, while others are during and after vaccination data, and regional and seasonal factors affecting the prevalence of *rotavirus* in Saudi Arabia. We found a reduction of *rotavirus* prevalence in Saudi Arabia before and after vaccination. This reduction might not only solely represent the impact of vaccination, but also other factors like improved sanitation, early diagnosis, proper water treatment, increased awareness and study reporting strategies might have contributed. The finding is similar to previous original studies in Saudi Arabia and other nearby countries in the Middle Eastern regions reporting a prevalence near 40% and in European children about 43% before vaccination (Arakaki et al., 2021; Damtie et al., 2020; Dey et al., 2020; Giri et al., 2018; Grimprel et al., 2008; Gupta et al., 2019; Güzel et al., 2020; Jagai et al., 2012; Jeong and Kim, 2014; Jesudason et al., 2024; Leshem et al., 2014; Monavari et al., 2017; Ouermi et al., 2017; Sadiq et al., 2019; Sharif et al., 2020a; Tcheremenskaia et al., 2007; Yu et al., 2019; Zhou et al., 2016). However, compared to other meta-analyses and surveillance studies conducted in LMICs like Bangladesh, Turkey, and China, we found a slightly higher prevalence of *rotavirus* among children under 5 years in Saudi Arabia (Arakaki et al., 2021; Damtie et al., 2020; Dey et al., 2020; Giri et al., 2018; Gupta et al., 2019; Güzel et al., 2020; Jeong and Kim, 2014; Jesudason et al., 2024; Leshem et al., 2014; Monavari et al., 2017; Sharif et al., 2020a; Yu et al., 2019; Zhou et al., 2016). We also found that the prevalence of *rotavirus* was higher than 10% in diarrheal children in Dammam and Makkah regions after vaccination. These data call for continuous monitoring of the sources of transmission of *rotavirus* in Saudi Arabia. Though the *rotavirus* vaccination program in Saudi Arabia is considered successful in reducing the burden of *rotavirus* diarrhea, reports of lower efficacy of *rotavirus* vaccines in developing countries and the emergence of non-included genotypes due to vaccine pressure can be future challenges. Second, we found the studies were concentrated on some fixed hospitals in selected regions of Dammam, Riyadh, Makkah, Jeddah, Jizan, Madinah, and Qassim. However, the lack of real-world community-based surveillance data is absent in Saudi Arabia. Among these, hospital-based surveillance, Dammam and Makkah were found to be heavily burdened with *rotavirus* compared to other regions in Saudi Arabia. We found lower heterogeneity of prevalence of *rotavirus* in Dammam and Makkah, Riyadh, Jeddah, and Najran after vaccination compared to the overall pooled prevalence. This might be due to the reduction of prevalence, reporting bias and lack of community-based studies. The probable disproportionate prevalence of *rotavirus* needs further investigation of the drinking water and food, and hygiene habits of the residents of these areas. In previous studies in India, Bangladesh, and China, it was found that contaminated water and foods serve as the main sources of *rotavirus* transmission and a higher burden of the disease (Dey et al., 2020; Giri et al., 2018; Jesudason et al., 2024; Ouermi et al., 2017; Sadiq et al., 2019; Tcheremenskaia et al., 2007). Third, we found a higher prevalence of co-circulating pathogens of diarrhea among children in Saudi Arabia. About 68% of the studies reported the presence of *adenovirus, norovirus*, pathogenic *E. coli, Salmonella* spp, and *Shigella* spp. Additionally, circulation of less-reported genotypes along with co-infection might pose a higher risk of diarrheal disease burden in the future. Compared to previous studies in developing and developed countries, we also found a similar prevalence of non-*rotavirus* diarrheal pathogens among the children (Dey et al., 2020, 2021; Giri et al., 2018; Jesudason et al., 2024; Ouermi et al., 2017; Sadiq et al., 2019; Sharif et al., 2020a,b; Tcheremenskaia et al., 2007). However, surveillance and epidemiologic studies are limited in Saudi Arabia compared to other developed and developing countries, which should be increased to reflect the pathogenic diversity after *rotavirus* vaccination.

Fourth, we found that all of the *rotavirus* cases in Saudi Arabia were associated with *rotavirus* A (100%). We also found a great diversity of genotypes among the circulating isolates. The vaccination event had significant effects in changing the frequency of circulating genotypes in Saudi Arabia. Overall, G1P[8], G2P[4], and G9P[8] showed higher prevalence compared to other genotypes. However, after the start of the vaccination program in 2013, the frequency of G1P[8] reduced, and G2P[4] increased in Saudi Arabia. Compared to previous studies, we found that G1P[8] was prevalent in Jordan and the United Arab Emirates, G2P[4] in Oman and Yemen, G2P[6] in Iraq, and G4P[8] in Lebanon before 2010. After the start of vaccination, G1P[8] and G9P[8] were the two major genotypes in the nearby countries, including Yemen, Lebanon, and Egypt (Jesudason et al., 2024). Based on the findings of the nearby countries, our findings also indicated that vaccine-driven selective pressure was one of the major contributors to genotypic shift in Saudi Arabia (Jesudason et al., 2024). Though the findings are similar to previous reports in the Middle East and other countries in South Asia, the prevalence of genotype G9P[8] was found to be significantly lower than in other countries (Arakaki et al., 2021; Giri et al., 2018; Gupta et al., 2019; Güzel et al., 2020; Monavari et al., 2017; Ouermi et al., 2017; Tcheremenskaia et al., 2007; Yu et al., 2019; Zhou et al., 2016). Among the other genotypes, G4P[8], G3P[8], and G12P[8] were found in consistent frequency before and after the vaccination program. The vaccine coverage in the majority of the regions is higher in Saudi Arabia. However, the genotypes G2P[4], G3P[8], G4P[8], and G12P[8] are circulating in relatively higher frequency than other G1 genotypes. This study found higher heterogeneity in the relative frequency of some of the genotypes across different studies. The probable underlying reasons might be the lack of continuous genotyping studies from similar regions, the lack of community-surveillance data, and the lack of a strong national surveillance system. The surveillance study in the northern regions is also lacking. The impact of vaccination and coverage frequency on the circulating genotypes is not well-studied in Saudi Arabia. This study suggests that due to the vaccination by Rotarix in the reported regions, the shifts in genotypes have occurred. These findings strongly resonate with previous findings in developed and developing countries (Arakaki et al., 2021; Damtie et al., 2020; Giri et al., 2018; Grimprel et al., 2008; Gupta et al., 2019; Güzel et al., 2020; Jagai et al., 2012; Jesudason et al., 2024; Leshem et al., 2014; Monavari et al., 2017; Ouermi et al., 2017; Sadiq et al., 2019; Tcheremenskaia et al., 2007; Yu et al., 2019; Zhou et al., 2016). Additionally, we found a higher prevalence of G1, G2, and G9 genotypes and among the P-types, P[8] and P[4] genotypes in Saudi Arabia. The incidence of G2 and G9 increased after vaccination, while the incidence of G1 reduced, which needs further investigation to elucidate the impact of vaccination on genotype distribution.

Fifth, the seasonality of *rotavirus* infection is well-studied in different regions of the world. However, the seasonality is not well explored in hot and arid regions. We found that *rotavirus* cases spiked during the winter season, with lower average daily temperatures in Saudi Arabia. This finding is strongly similar to the previous findings globally (Jagai et al., 2012; Sharif et al., 2023). Additionally, we found *rotavirus* infection had higher odds during the winter in Saudi Arabia. However, some of the included works mentioned higher incidences during June to September with higher day temperatures. These data call for further investigation of the seasonal effects of *rotavirus* transmission in Saudi Arabia.

We found that *rotavirus*-positive children suffered from different clinical presentations (Afifi and Nabiha, 2013; Al-Ayed et al., 2017; Almalki et al., 2022; Alqurayn et al., 2024; Aly et al., 2015; Eifan et al., 2023; El Assouli et al., 1996; Ghazi et al., 2005; Hegazi et al., 2017; Khalil et al., 2015; Kheyami et al., 2008; Meqdam and Thwiny, 2007; Obeid, 2011; Paul et al., 2021; Qadri et al., 1990; Tayeb et al., 2008; Tayeb et al., 2011; Zaki et al., 2017). *Rotavirus*-positive children had significantly higher odds of severe diarrhea, vomiting, and fever in Saudi Arabia. These findings are similar to previous studies in Saudi Arabia and other developing countries (Arakaki et al., 2021; Grimprel et al., 2008; Gupta et al., 2019; Güzel et al., 2020; Jagai et al., 2012; Leshem et al., 2014; Monavari et al., 2017; Ouermi et al., 2017; Sharif et al., 2023; Tcheremenskaia et al., 2007; Yu et al., 2019; Zhou et al., 2016). Among other factors, children aged <2 years had significantly higher odds of *rotavirus* infection with severe health outcomes. There might be several reasons, including children aged >2 years already having antibodies against *rotavirus* due to previous infection and stronger immune systems compared to children aged <2 years. Further, the odds of prevalence of *rotavirus* infection reduced significantly after implementation of vaccination, which strongly resonates with previous studies in developed countries.

There are a number of limitations in this study that can be addressed in the future. Firstly, we could not add any *rotavirus* vaccination data due to the unavailable of studies. The findings are reported from hospital-based surveillance that may not truly represent the overall community prevalence. To get a more accurate insight into the epidemiology and genetic diversity of *rotaviruses*, community-based surveillance and hospital surveillance should be combined in the future. Further, we found genotypic data only from seven studies that might underscore the actual diversity in Saudi Arabia. More inclusive studies involving a higher number of samples from community-based surveillance should be conducted to reflect the circulating genotypes in the future. Moreover, phylogenetic and mutational analysis of the isolated genotypes could not be conducted in this study. In addition, the diagnostic methods reported in different papers were either ELISA or RT-PCR. However, we found minimal heterogeneity in regarding the used diagnostic methods. It should be considered with more strict approach in the future studies. In the future, more data from all over Saudi Arabia should be included to create a complete scenario of *rotavirus*-associated health burden.

## Conclusion

This is one of the first meta-analyses including the majority of the studies from 1985 to 2025 in Saudi Arabia. This study will provide an integrated insight into *rotavirus* epidemiology in Saudi Arabia. We found a higher prevalence of *rotavirus* before vaccination, and after the start of vaccination, it decreased significantly from 35% to about 2% in some of the regions in Saudi Arabia. However, in some of the regions, the prevalence is still higher than 10%. We also found genotypes G2P[4], and G9P[8] became prevalent over G1P[8] after vaccination. This study calls for more epidemiologic and genotypic analysis of *rotavirus*, expanding to all of regions in Saudi Arabia.

## Data Availability

The raw data supporting the conclusions of this article will be made available by the authors, without undue reservation.

## Footnotes

1
https://weather.kau.edu.sa/Default-155001-EN

2
https://www.weather-atlas.com/en/saudi-arabia

3
www.accuweather.com

4
www.meteoblue.com

5
www.weatheronline.co.uk
